# Postnatal dexamethasone, respiratory and neurodevelopmental outcomes at two years in babies born extremely preterm

**DOI:** 10.1371/journal.pone.0181176

**Published:** 2017-07-19

**Authors:** Gordon Qin, Jessica W. Lo, Neil Marlow, Sandy A. Calvert, Anne Greenough, Janet L. Peacock

**Affiliations:** 1 Division of Health and Social Care Research, King’s College London, London, United Kingdom; 2 Queen Alexandra Hospital, Portsmouth, Hampshire, United Kingdom; 3 School of Psychiatry, UNSW Medicine, University of New South Wales, Randwick, Australia; 4 Institute for Women’s Health, University College London, London, United Kingdom; 5 Department of Child Health, St George’s University of London, London, United Kingdom; 6 Division of Asthma, Allergy and Lung Biology, King's College London, London, United Kingdom; University of Giessen Lung Center, GERMANY

## Abstract

**Importance:**

Postnatal dexamethasone is associated with reduction in bronchopulmonary dysplasia. There remains, however, concern that its short-term benefits are accompanied by long-term adverse effects e.g. poorer neurodevelopmental outcomes.

**Objective:**

Our aim was to determine the effects of administration of postnatal dexamethasone on respiratory and neurodevelopmental outcome at two years of age after adjusting for neonatal and infant risk factors.

**Materials and methods:**

The study included 412 infants born at 23–28 weeks of gestation, 29% had received postnatal dexamethasone. Two outcomes were examined, respiratory hospital admissions in the past 12 months and neurodevelopmental impairment. Logistic regression, adjusted for sex, birthweight z-score, gestation, maternal smoking, oxygen dependency at 36 weeks, airleak, patent ductus arteriosus, pulmonary haemorrhage, major ultrasound abnormality, mode of ventilation and age at assessment, was undertaken.

**Results:**

After adjustment, postnatal dexamethasone was associated with significantly increased proportions of both respiratory hospital readmission: (0.35 vs 0.15, difference = 0.20; 95% CI: 0.08, 0.31) and neurodevelopmental impairment (0.59 vs 0.45, difference = 0.14; 95% CI: 0.02, 0.26).

**Conclusions:**

Postnatal dexamethasone use in extremely preterm infants is associated with increased risks of respiratory hospital admissions and neurodevelopmental impairment. These associations were not explained by excess neonatal morbidities.

## Introduction

Infants born prematurely usually require respiratory support which, although often essential for survival, can lead to lung damage, prolonged oxygen dependency and chronic respiratory morbidity. The administration of the corticosteroids can improve respiratory function and allow earlier extubation. Systematic review of randomised trials demonstrated that the administration of steroids before seven days facilitated extubation and reduced bronchopulmonary dysplasia (BPD) [1]. Delayed corticosteroid treatment, i.e. after seven days, was also associated with reductions in failure to extubate and BPD [2]. Corticosteroids, however, are linked to adverse neurological effects when given early [1, 3] or late [2] and linked to inhibited secondary septation [4].

The aim of this study was to use the neonatal and two-year follow-up data from the United Kingdom Oscillation Study (UKOS) [5,6] to explore the relationship between the use of postnatal dexamethasone and later outcomes. We wished to determine whether there was any association with long term respiratory morbidity, as indicated by respiratory hospital readmission and if in the UKOS extremely, prematurely born cohort there was evidence of increased abnormal neurological outcome, as had been previously reported [7,8]. Furthermore, we investigated whether any late adverse effects observed were explained by other neonatal morbidities and if they correlated with the number of courses received.

## Methods

**Fig 1:**
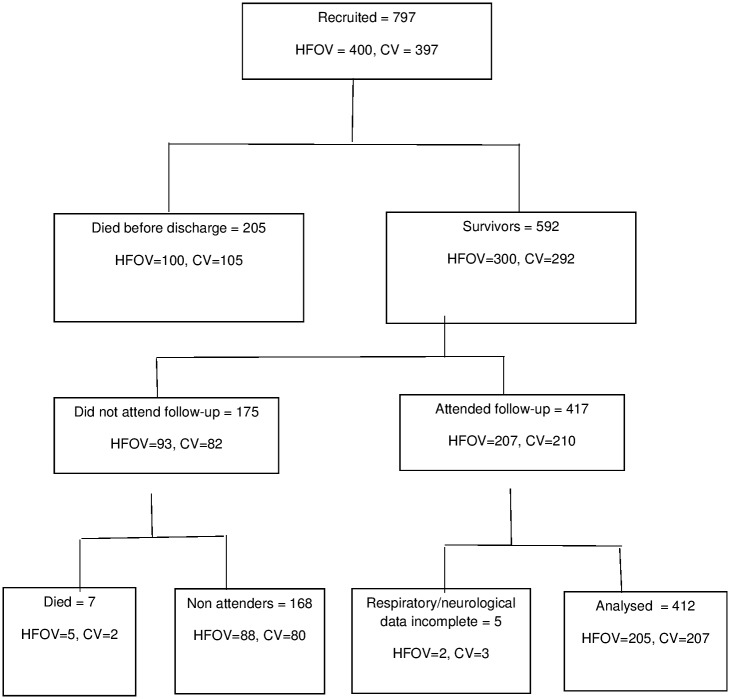
Study flow chart.

In the UKOS trial, 797 babies were randomised to either high frequency oscillatory ventilation (HFOV) or conventional ventilation (CV) [5]. Five hundred and ninety-two infants survived to hospital discharge; the 412 infants who were seen at two years of age formed this dataset (Fig 1). The UKOS trial and follow-up were approved by the London South Thames Multicentre Research Ethics Committee. Parents gave informed written consent for their infants to take part.

The administration of postnatal dexamethasone was assessed as i) received postnatal dexamethasone yes/no, ii) the timing of the dexamethasone administration (≤7 days = Early/>7 days = Late) using the definitions from the Cochrane reviews [1,2], iii) the number of courses received, and iv) the number of days of dexamethasone exposure (analysed in four groups: 0 days, 1–6 days, 7–12 days and >12 days). UKOS participating centres were surveyed and confirmed the postnatal steroid used was dexamethasone.

Respiratory outcome at age two was determined as any respiratory hospital admission in the previous 12 months as reported by parents to the paediatrician in a structured interview when their child was two years corrected age [6]. To determine whether a respiratory hospital admission, as recalled by the parents, was a true reflection of respiratory morbidity we assessed whether admissions were related to reported cough or wheeze or use of ‘chest’ medication as recorded at the same time, two years of age. Neurological outcome at age two years corrected was defined in the same way as in our main UKOS follow-up paper [6] as at least one abnormality in one of the following domains: neuro-motor, vision, hearing, communication or other physical disabilities, such as cerebral palsy. No impairment was defined as ‘normal’, as was a missing response to all of the above domains. This reflected clinical opinion as to the meaning of ‘missing’ in this context. [6] These two outcomes, respiratory admissions and neurodevelopmental impairment were chosen *a priori* as indicators of morbidity. No other analyses were performed.

### Analysis

In the primary analysis we used multivariable logistic regression to examine the effect of postnatal dexamethasone on the above outcomes, adopting a similar analysis strategy to that we have used in earlier work in the UKOS study [9] and in other research in the long-term effects of postnatal dexamethasone [10]. The following neonatal factors were included as potential associations of poor outcome at age two: infant sex, birthweight z-score, gestational age, maternal smoking during pregnancy, oxygen dependency at 36 weeks postmenstrual age (PMA), major cranial ultrasound abnormality, airleak, patent ductus arteriosus (PDA), pulmonary haemorrhage and mode of ventilation. Age at the two year assessment was also included as a potential confounder. The association between each postnatal dexamethasone measure and each outcome was first analysed in the regression model without adjustment and then with adjustment for confounders. In a sensitivity analysis, we adjusted for oxygen dependency at 28 days in place of oxygen dependency at 36 weeks PMA.

Since the use of postnatal dexamethasone is so highly confounded by neonatal factors, we also used propensity score matching for dexamethasone exposure (yes/no) [11] as an alternative way of adjustment in addition to multivariable logistic regression. Propensity score (PS) matching works differently to multiple logistic regression by matching the subjects as closely as possible using baseline factors prior to analysis so that the study closely resembles a randomised trial. The following baseline factors were used in the propensity score matching: sex, birthweight, birthweight z-score, gestational age in weeks, smoking in pregnancy, multiple birth, ventilation group and Apgar score. It is not recommended to adjust for non-baseline factors and so age at assessment was not used in the PS analysis. The main challenge of the PS method is to obtain close matches for all subjects. It was not possible to use propensity score matching for three measures of dexamethasone exposure, that is timing of administration, number of courses and days of exposure due to the small numbers in the different dexamethasone-use categories. For this reason only adjustment by multivariable logistic regression was undertaken for those measures. The effects of postnatal dexamethasone were calculated as odds ratios but reported in the main text as the marginal proportions in the categories and differences with 95% confidence interval to improve clinical interpretability. Results as odds ratios are given in the supporting information (Tables A,B in S1 File). Marginal proportions could not be calculated for the propensity score estimates and so the odds ratios for the propensity score analyses (OR) were converted to a difference in proportions (p1-p2) as follows: using the observed proportion for the unexposed group (p1) then calculate the calculate the proportion in the exposed group (p2) using the formula: p2 = p1/(OR-OR*p1+p1).

Finally we computed the correlations between respiratory admissions and respiratory symptoms and present these as odds ratios to validate the use of respiratory admissions as a summary of poor respiratory outcome.

Each analysis used cases with complete data so that the numbers were the same for each analysis. All statistical analyses were performed using the Stata 14 software.

## Results

The baseline characteristics of those with and without complete data were similar apart from oxygen dependency at 36 weeks postmenstrual age (PMA) which was commoner in those with complete data, 58% vs 49% (Table 1). This was true in those with and without exposure to postnatal dexamethasone: 82% vs 75% were oxygen dependent at 36 weeks PMA among those exposed compared to 49% vs 39% in the unexposed. Oxygen dependency at 36 weeks PMA was included in both the multivariable analyses and the propensity score matching.

**Table 1:** Baseline characteristics of the infants included in this study and those not included, based on incomplete data for postnatal dexamethasone use and age 2 outcomes as defined.

	Sample analysed with complete data N = 412	Sample not analysed with incomplete data N = 180	Comparison of complete and incomplete samples
Characteristics	% (n) or mean (SD)	% (n) or mean (SD)	p-value
Birth weight	899 (210)	907 (193)	0.66
Gestational age	26.4 (1.3)	26.4 (1.3)	0.88
Male sex	52% (216)	52% (93)	0.87
Multiple birth	23% (96)	19% (35)	0.30
Postnatal dexamethasone Use	29% (120)	30% (52)	0.85
Oxygen dependency at 28 days	81% (335)	77% (139)	0.25
Oxygen dependency at 36 weeks PMA	58% (240)	49% (88)	0.04
Oxygen dependency at hospital discharge	23% (93)	16% (29)	0.08
Major ultrasound abnormality in neonatal period	14% (59)	13% (23)	0.63
Maternal smoking in pregnancy	25% (99)	33% (52)	0.07

One hundred and twenty out of 412 infants (29%) had received postnatal dexamethasone, of those 63 (53%) received one course, 26% received two courses and the remainder received three courses. The majority (93%) had received dexamethasone more than seven days after their birth. Forty three percent of infants had received dexamethasone for more than 12 days, 37% for 7–12 days and the remainder for less than 7 days.

Eighty-eight (21%) of the infants had a respiratory hospital readmission between age one and two years. Postnatal dexamethasone exposure was associated with a statistically significant increased proportion of readmissions in the previous 12 months both before and after statistical adjustment (0.35 vs 0.15, Table 2). This is equivalent to a number needed to treat of 5. The difference in proportions was reduced in size with propensity score matching, but it remained statistically significant (difference = 0.03 (95% CI 0.01, 0.06), p = 0.005). The risk of hospital admission was positively associated with increasing numbers of courses of dexamethasone and this remained significant after adjustment. Both early and late administration of dexamethasone was significantly associated with increased proportions of respiratory hospital admissions in comparison to no exposure, both before and after adjustment. Additionally, the difference in proportions was reduced in size for the late administration of dexamethasone in comparison to early (Table 2). Respiratory hospital admission was strongly associated with cough (OR = 4.44; 95% CI: 2.60, 7.57), wheeze (5.15; 3.07, 8.64) and use of chest medication (10.38; 4.93, 21.83) at two years of age.

**Table 2:** Respiratory hospital admissions and postnatal dexamethasone exposure.

	Respiratory hospital admission n = 88, 21%		
	N	Unadjusted proportions	Adjusted proportions ^1^
**Dexamethasone exposure**		p-value<0.001	
**No**	292	0.15	0.15
**Yes**	120	0.36	0.35
**Difference (95% CI)**			
**Yes vs no**		0.20 (0.11, 0.30)	0.20 (0.08, 0.31)
**Timing of dexamethasone**		Overall p-value: <0.001	Overall p-value: <0.001
**None**	292	0.15	0.15
**Early**	9	0.44	0.48
**Late**	111	0.35	0.34
**Differences (95% CI)**			
**Early vs none**		0.29 (-0.04, 0.62)	0.33 (-0.01, 0.66)
**Late vs none**		0.20 (0.10, 0.30)	0.18 (0.07, 0.30)
**No. of courses**		Overall p-value: <0.001	Overall p-value: 0.001
**0 course**	292	0.15	0.15
**1 course**	76	0.34	0.33
**2 courses**	31	0.39	0.38
**3 courses**	13	0.38	0.40
**Differences (95% CI)**			
**1 vs 0 courses**		0.19 (0.07, 0.30)	0.18 (0.06, 0.30)
**2 vs 0 courses**		0.23 (0.06, 0.41)	0.23 (0.03, 0.43)
**3 vs 0 courses**		0.23 (-0.04, 0.50)	0.25 (-0.05, 0.55)
**No. of days of exposure**		Overall p-value: <0.001	Overall p-value: <0.001
**0**	292	0.15	0.15
**1–6**	24	0.21	0.22
**7–12**	44	0.41	0.39
**>12**	51	0.40	0.38
**Differences (95% CI)**			
**1–6 vs 0**		0.05 (-0.11, 0.22)	0.07 (-0.10, 0.24)
**7–12 vs 0**		0.25 (0.10, 0.41)	0.23 (0.08, 0.39)
**>12 vs 0**		0.24 (0.10, 0.38)	0.23 (0.07, 0.39)

^1^. Adjusted for sex, birthweight z-score, gestational age in weeks, smoking in pregnancy, oxygen dependency at 36 weeks, airleak, patent ductus arteriosus, pulmonary haemorrhage, major neonatal ultrasound abnormality, ventilation group and age at 24 month assessment using multiple logistic regression analysis.

Overall 201 (49%) of the infants had some neurodevelopmental impairment at age two. Postnatal dexamethasone exposure was associated with a significantly increased proportion of any impairment before adjustment and, although the difference in proportions was reduced in size after adjustment, it remained statistically significant (Table 3). This association was confirmed with the propensity score matching which also indicated a significantly increased proportion of any impairment with postnatal dexamethasone exposure (difference = 0.07; 95%CI: 0.03,0.11, p < 0.001). The association between the number of courses of dexamethasone and any impairment remained significant after adjustment while there was no clear association of early administration of dexamethasone on impairment although numbers were small n = 9, Table 3).

**Table 3:** Any neurodevelopmental impairment and postnatal dexamethasone exposure.

	Any neurodevelopmental impairment n = 201 49%		
	N	Unadjusted proportions	Adjusted proportions ^1^
**Dexamethasone exposure**		p-value<0.001	
**No**	292	0.43	0.45
**Yes**	120	0.63	0.59
**Difference (95% CI)**			
**Yes vs no**		0.19 (0.09, 0.30)	0.14 (0.02, 0.26)
**Timing of dexamethasone**		Overall p-value: <0.001	Overall p-value: <0.001
**None**	292	0.43	0.45
**Early**	9	0.56	0.56
**Late**	111	0.63	0.59
**Differences (95% CI)**			
**Early vs none**		0.12 (-0.21, 0.45)	0.11 (-0.22, 0.45)
**Late vs none**		0.20 (0.09, 0.31)	0.14 (0.02, 0.27)
**No. of courses**		Overall p-value: <0.001	Overall p-value: 0.001
**0 course**	292	0.43	0.45
**1 course**	76	0.62	0.59
**2 courses**	31	0.61	0.58
**3 courses**	13	0.69	0.57
**Differences (95% CI)**			
**1 vs 0 courses**		0.19 (0.06, 0.31)	0.14 (0.01, 0.28)
**2 vs 0 courses**		0.18 (0.00, 0.36)	0.13 (-0.06, 0.32)
**3 vs 0 courses**		0.26 (0.00, 0.52)	0.12 (-0.21, 0.45)
**No. of days of exposure**		Overall p-value: <0.001	Overall p-value: <0.001
**0**	292	0.43	0.45
**1–6**	24	0.75	0.70
**7–12**	44	0.50	0.45
**>12**	51	0.67	0.65
**Differences (95% CI)**			
**1–6 vs 0**		0.32 (0.14, 0.50)	0.25 (0.06, 0.45)
**7–12 vs 0**		0.07 (-0.09, 0.23)	0.00 (-0.18, 0.18)
**>12 vs 0**		0.24 (0.09, 0.38)	0.21 (0.05, 0.37)

^1^. Adjusted for sex, birthweight z-score, gestational age in weeks, maternal smoking in pregnancy, oxygen dependency at 36 weeks PMA, airleak, patent ductus arteriosus, pulmonary haemorrhage, major neonatal ultrasound abnormality, ventilation group and age at 24 month assessment using multiple logistic regression analysis.

The final sensitivity analysis for the regression models: adjusted for oxygen dependency at 28 days in place of oxygen dependency at 36 weeks (Table C in S1 File) showed very similar results to those described above. The results expressed as odds ratios are shown in tables A and B of the supporting information document (Table A, B in S1 File).

Diagnostic investigation of the propensity score matching showed that all cases were matched and that data from only two subjects were more than 0.025 apart from their match. Sensitivity analysis excluding these two subjects made no appreciable difference to the estimates. These results and the distribution of the propensity scores are shown in the supporting information (Figure A, Text, Table A in S1 File).

## Discussion

We have shown an association between exposure to early or late postnatal dexamethasone and respiratory hospital readmission. This association was reduced but remained statistically significant after adjustment for neonatal and infant factors and was robust to adjustment using two different statistical approaches. The proportion of infants who had had respiratory admissions increased with the number of courses. The associations with respiratory admissions remained statistically significant after adjustment using propensity score matching. We observed strong correlations between reported respiratory admission and cough, wheeze and use of chest medications, validating the use of respiratory admission as a marker of respiratory morbidity.

The results of studies examining respiratory health in childhood after postnatal steroid administration have been conflicting. In one series, children who had received postnatal corticosteroid had lower FEV_1_ (82 vs 88%, p = 0.006) and FEF_25-75_ (65% versus 78%, p = 0.003). The mean cumulative dose of postnatal corticosteroid administration was 7.8 mg/kg, but no dose effect on lung function was found [12]. In the largest follow-up study, Jones [13] evaluated pulmonary function in 142 children aged 13 to 17 years who participated as infants in the Collaborative Dexamethasone Trial [14], a multicentre postnatal randomized controlled trial of one week of dexamethasone treatment. Even though beneficial effects of dexamethasone were seen in the postnatal period (i.e. fewer days of ventilator dependence) [15] no differences between the dexamethasone (n = 68) and placebo (n = 74) groups were noted at follow-up in terms of FVC, FEV_1_, FEV_1_/FVC, FEF_25-75_, peak expiratory flow rate, bronchodilator responsiveness, or respiratory morbidity (ie, current asthma, wheezing, coughing, inhaler use). It is important to note that a large proportion of the placebo group (39%) were exposed to open-label dexamethasone which may have attenuated possible treatment effects. A study pulmonary function at 15 years of age was significantly better for children who had received a 42 day course of postnatal dexamethasone compared to an 18 day course (FEV_1_, 90% versus 71%) and there was no significant difference in the lung function of the 42 day group and the controls [16]. In that study, the children who received a 42 day course had a sustained improvement in respiratory health and neurodevelopment, whereas those receiving the 18 day course after an initial improvement required increased respiratory support once the steroids were discontinued. Another study [17] demonstrated less dexamethasone treated infants at 8–11 years of age had an FEV_1_ below normal (40% versus 68%, p = 0.03), but parent reported prevalence of asthma did not differ between the two groups. Logistic regression analysis suggested the positive effects of dexamethasone were mediated in part by a shortened postnatal exposure to mechanical ventilation. The infants had received a 42 day tapering course of steroids [17]. Mieskonen et al also showed a beneficial effect of postnatal dexamethasone but their sample size was small, eight were treated with a week long course of dexamethasone and eight given placebo. They found a significantly higher FVC in 7–9 year old children but no differences in forced expiratory flow rate [18]. Of all the above studies, only the subjects of Smith et al [12] were born in the post-surfactant era and were generally born more prematurely than the participants in the other studies. Their population [12] then are the most similar to our own, both studies demonstrated in infants routinely exposed to antenatal steroids and postnatal surfactant, postnatal steroids were associated with worse long term respiratory outcomes.

This follow-up study in infants born extremely prematurely has shown an association between both early and late administration of postnatal dexamethasone and poor neurodevelopmental outcome at age two years. This association was reduced in strength but remained statistically significant after adjustment for neonatal and infant factors and as with respiratory admission, the association remained significant after adjustment using two different statistical approaches. Dexamethasone has been associated with altered maturation of neurones in preterm infants which may account for the adverse neurodevelopmental effects of postnatal treatment [7]. Other possible mechanisms for reduced brain growth associated with postnatal dexamethasone include inhibition of growth factors and facilitation of apoptosis [7]. In a previous study [10] neurodevelopmental impairment was also increased with increasing dose of dexamethasone, but not postnatal age.

This study has strengths and some limitations. The data were from a randomised trial [5]; there were no significant differences shown in the short term outcomes [6] and adjusting for the intervention group did not affect the results presented in this paper. The baseline characteristics in those with and without complete data were similar apart from oxygen dependency at 36 weeks postmenstrual age which was commoner in those with complete data. This is consistent with our previous observations that children with missing data and those who dropped out tend to have fewer health problems. This observation held in both those with and without exposure to postnatal dexamethasone. This was included in the multivariable analyses and the propensity score matching. Further, sensitivity analyses using 28-day oxygen dependency showed similar results and so overall we do not consider that this has biased the results presented here.

In conclusion, our data provide evidence that in extremely premature infants, the use of postnatal dexamethasone is associated with an increased risk of respiratory morbidity and neurodevelopmental impairment at follow up. These associations were not explained by a higher proportion of infants with neonatal morbidities in the dexamethasone group. In addition, the risk of respiratory related hospital admission was increased by increasing numbers of postnatal dexamethasone courses.

## Supporting information

S1 File Supporting information. (PDF)

## Abbreviations

CV: Conventional ventilation
FEF_25-75_: Forced expiratory flow between 25 and 75%
FEV_1_: Forced expiratory volume in one second
FVC: Forced vital capacity
HFOV: High frequency oscillatory ventilation
OR: Odds ratio
PDA: Patent ductus arteriosus
PMA: Postmenstrual age
PS: Propensity score
UKOS: United Kingdom Oscillation Study
