# Supplementary material for: Postnatal dexamethasone, respiratory and neurodevelopmental outcomes at two years in babies born extremely preterm
Source: PLoS One. 2017 Jul 19;12(7):e0181176. doi: 10.1371/journal.pone.0181176 (PMC5517008; doi:10.1371/journal.pone.0181176)
Supplement: S1 File — (PDF) [file pone.0181176.s001.pdf]

Supporting information (S1)

Postnatal Dexamethasone, respiratory and neurodevelopmental outcomes at two years in babies born extremely preterm

Gordon Qin, Jessica W Lo, Neil Marlow, Sandy A Calvert, Anne Greenough, Janet L Peacock

Page	Item
2	Table A Respiratory hospital admissions and dexamethasone exposure expressed as odds ratios (see table 2 in main text for equivalent analyses as proportions)
3	Table B. Any neurodevelopmental impairment and postnatal steroid exposure expressed as odds ratios (see table 3 in main text for equivalent analyses as proportions)
4	Table C. Sensitivity analysis of the multivariable regression model using oxygen dependency at 28 days instead of oxygen dependency at 36 weeks PMA
5	Further information on propensity score matching

Table A Respiratory hospital admissions and dexamethasone exposure expressed as odds ratios (see table 2 for equivalent analyses as proportions)

Respiratory hospital admission n=88, 21%		
	**Unadjusted**	**Adjusted[1]**
	**Odds ratio (95% CI)**<br>**p-value**	**Odds ratio (95% CI)**<br>**p-value**
**Dexamethasone exposure**		
**No**	1.00	1.00
**Yes**	3.07 (1.88 – 5.00)<br><0.001	3.14 (1.69 – 5.84)<br><0.001
**Timing of dexamethasone**	Overall p-value:<br><0.001	Overall p-value:<br><0.001
**None**	1.00	1.00
**Early**	4.39 (1.14 – 17.0)<br>0.032	5.56 (1.21 – 25.6)<br>0.027
**Late**	2.97 (1.80 – 4.92)<br><0.001	2.96 (1.56 – 5.59)<br>0.027
**No. of courses**	Overall p-value:<br><0.001	Overall p-value:<br>0.001
**0 course**	1.00	1.00
**1 course**	2.85 (1.61 – 5.05)	2.90 (1.47 – 5.73)
**2 courses**	3.47(1.57 – 7.63)	3.66 (1.42 – 9.44)
**3 courses**	3.43 (1.07 – 11.0)	4.02 (1.07 – 15.1)
**No. of days of exposure**	Overall p-value:<br><0.001	Overall p-value:<br><0.001
**0**	1.00	1.00
**1-6**	1.44 (0.51 – 4.07)	1.60 (0.52 – 4.95)
**7-12**	3.80 (1.92 – 7.50)	3.73 (1.69 – 8.23)
**>12**	3.54 (1.86 – 6.75)	3.66 (1.66 – 8.05)

Footnotes: 1. Adjusted for sex, birthweight z-score, gestational age in weeks, smoking in pregnancy, oxygen dependency at 36 weeks, airleak, patent ductus arteriosus, pulmonary haemorrhage, major neonatal ultrasound abnormality, ventilation group and age at 24 month assessment using multiple logistic regression analysis.

Table B. Any neurodevelopmental impairment and postnatal steroid exposure expressed as odds ratios (see table 3 for equivalent analyses as proportions)

**Any neurodevelopmental impairment n=201, 49%**		
	**Unadjusted**	**Adjusted [1]**
	**Odds ratio (95% CI)**<br>**p-value**	**Odds ratio (95% CI)**<br>**p-value**
**Dexamethasone exposure**		
**No**	1.00	1.00
**Yes**	2.20 (1.42 – 3.40)<br><0.001	1.88 (1.08 – 3.28)<br>0.026
**Timing of dexamethasone**	Overall p-value:<br><0.001	Overall p-value:<br>0.026
**None**	1.00	1.00
**Early**	1.65 (0.43 – 6.26)<br>0.46	1.66 (0.39 – 7.12)<br>0.49
**Late**	2.25 (1.43 – 3.53)<br><0.001	1.91 (1.07 – 3.40)<br>0.028
**No. of courses**	Overall p-value:<br>0.001	Overall p-value:<br>0.074
**0 course**	1.00	1.00
**1 course**	2.14 (1.27 – 3.58)	1.93 (1.03 – 3.62)
**2 courses**	2.09 (0.98 – 4.46)	1.81 (0.74 – 4.43)
**3 courses**	2.96 (0.89 – 9.85)	1.72 (0.42 – 7.00)
**No. of days of exposure**	Overall p-value:<br>0.002	Overall p-value:<br>0.04
**0**	1.00	1.00
**1-6**	3.95 (1.52 – 10.2)	3.26 (1.14 – 9.30)
**7-12**	1.32 (0.70 – 2.49)	0.99 (0.46 – 2.16)
**>12**	2.63 (1.41 – 4.93)	2.60 (1.20 – 5.57)

Footnotes: 1. Adjusted for sex, birthweight z-score, gestational age in weeks, maternal smoking in pregnancy, oxygen dependency at 36 weeks PMA, airleak, patent ductus arteriosus, pulmonary haemorrhage, major neonatal ultrasound abnormality, ventilation group and age at 24 month assessment using multiple logistic regression analysis.

Table C. Sensitivity analysis of the multivariable regression model using oxygen dependency at 28 days instead of oxygen dependency at 36 weeks PMA

	Outcome: Respiratory hospital admissions	Outcome: Any impairment
	Odds ratio (95% CI)<br>p-value	Odds ratio (95% CI)<br>p-value
**Dexamethasone exposure**		
**No**	1.00	1.00
**Yes**	3.19 (1.73 – 5.89)<br><0.001	1.87 (1.08 – 3.25)<br>0.026
**Timing of dexamethasone**	Overall p-value:<br><0.001	Overall p-value:<br>0.027
**None**	1.00	1.00
**Early**	5.67 (1.24 – 26.0)<br>0.026	1.67 (0.39 – 7.16)<br>0.49
**Late**	3.00 (1.60 – 5.64)<br>0.001	1.89 (1.07 – 3.36)<br>0.028
**No. of courses**	Overall p-value:<br>0.001	Overall p-value:<br>0.071
**0 course**	1.00	1.00
**1 course**	2.93 (1.49 – 5.76)	1.91 (1.02 – 3.59)
**2 courses**	3.73 (1.46 – 9.54)	1.80 (0.74 – 4.39)
**3 courses**	4.17 (1.12 – 15.5)	1.75 (0.44 – 7.06)
**No. of days of exposure**	Overall p-value:<br><0.001	Overall p-value:<br>0.038
**0**	1.00	1.00
**1-6**	1.59 (0.51 – 4.92)	3.24 (1.13 – 9.25)
**7-12**	3.74 (1.70 – 8.23)	0.98 (0.45 – 2.11)
**>12**	3.73 (1.72 – 8.10)	2.63 (1.24 – 5.60)

Footnotes: 1. Adjusted for sex, birthweight z-score, gestational age in weeks, maternal smoking in pregnancy, oxygen dependency at 28 days, airleak, patent ductus arteriosus, pulmonary haemorrhage, major neonatal ultrasound abnormality, ventilation group and age at 24 month assessment using multiple logistic regression analysis.

Further information on propensity score matching

Figure A: distribution of propensity scores by postnatal steroids given ('syster'=yes/no)

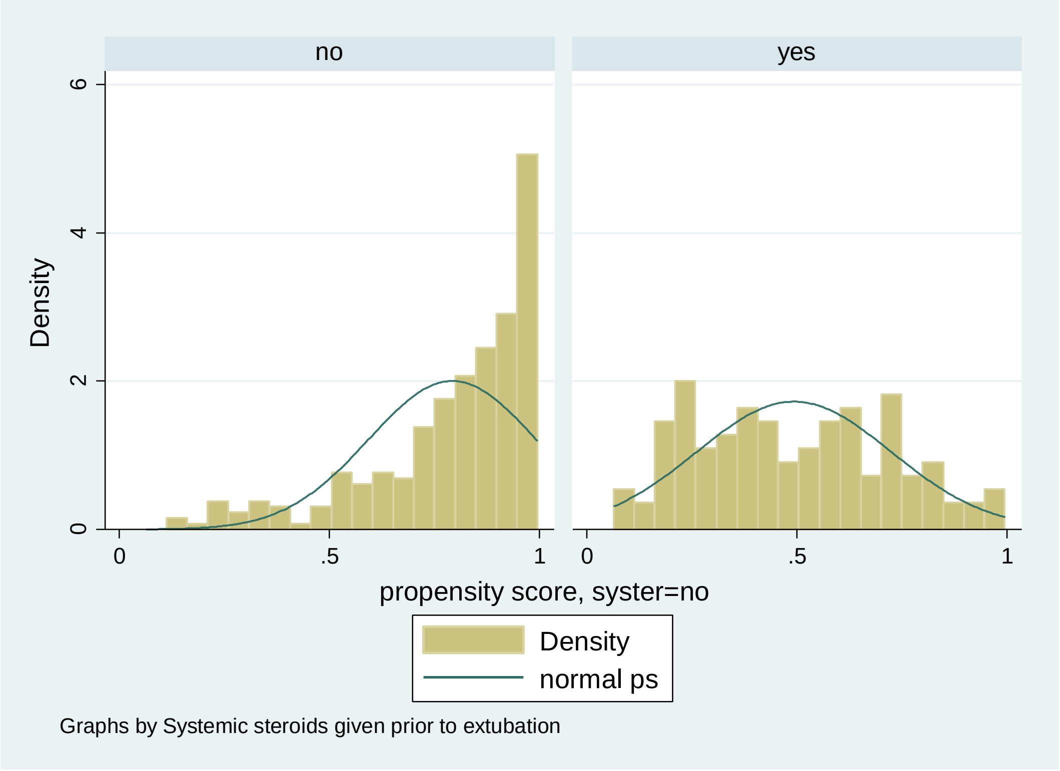

Matching

Full matching was achieved in the analysis with just two observations not achieving a match within 0.025. A sensitivity analysis of without these two subjects gave a very similar result for each of the two outcomes as shown below

Model	OR	95% CI	P
Respiratory hospital admissions all subjects	1.26	1.07, 1.49	0.005
Respiratory hospital admissions Without 2 subjects with poorer match	1.24	1.05, 1.47	0.010
Neurodevelopmental impairment: all subjects	1.34	1.15, 1.58	<0.001
Neurodevelopmental impairment: without 2 subjects with poorer match	1.36	1.16, 1.59	<0.001
